# Leprosy on Reunion Island, 2005-2013: Situation and Perspectives

**DOI:** 10.1371/journal.pntd.0004612

**Published:** 2016-04-15

**Authors:** Guillaume Camuset, Sophie Lafarge, Gianandrea Borgherini, Anne Gerber, Nicolas Pouderoux, Aurélie Foucher, Patrice Poubeau, Rodolphe Manaquin, Sophie Larrieu, Pascal Vilain, Laetitita Huiart

**Affiliations:** 1 Centre Hospitalier Universitaire de la Réunion, Department of Infectious Disease, Saint-Pierre, France; 2 Centre Hospitalier Universitaire de la Réunion, INSERM, CIC 1410, Saint-Pierre, France; 3 Centre Hospitalier Universitaire de la Réunion, Department of Internal Medicine, Saint-Denis, France; 4 Centre de Lutte Anti-tuberculeuse Nord et Est, Saint-Denis, France; 5 Regional Office of French Institute for Public Health Surveillance of Indian Ocean, Saint-Denis, France; Hospital Infantil de Mexico Federico Gomez, UNITED STATES

## Abstract

**Background:**

Reunion Island is a French overseas territory located in the south-western of Indian Ocean, 700 km east of Madagascar. Leprosy first arrived on Reunion Island in the early 1700s with the African slaves and immigration from Madagascar. The disease was endemic until 1980 but improvement of health care and life conditions of inhabitants in the island have allowed a strong decrease in new cases of leprosy. However, the reintroduction of the disease by migrants from endemic neighbouring countries like Comoros and Madagascar is a real and continuing risk. This observational study was then conducted to measure the number of new cases detected annually on Reunion Island between 2005 and 2013, and to describe the clinical features of these patients.

**Methodology/Principal Findings:**

Data were collected over two distinct periods. Incident cases between 2005 and 2010 come from a retrospective study conducted in 2010 by the regional Office of French Institute for Public Health Surveillance (CIRE of Indian Ocean), when no surveillance system exist. Cases between 2011 and 2013 come from a prospective collection of all new cases, following the implementation of systematic notification of all new cases. All patient data were anonymized. Among the 25 new cases, 12 are Reunion Island residents who never lived outside Reunion Island, and hence are considered to be confirmed autochthonous patients. Registered prevalence in 2014 was 0.05 /10 000 habitants, less than the WHO’s eradication goal (1/10 000).

**Conclusions/Significance:**

Leprosy is no longer a major public health problem on Reunion Island, as its low prevalence rate indicates. However, the risk of recrudescence of the disease and of renewed autochthonous transmission remains real. In this context, active case detection must be pursued through the active declaration and rapid treatment of all new cases.

## Author Summary

Leprosy was still endemic on Reunion Island 30 years ago but improvements in health care and treatments led to a significant decrease in the number of new cases of leprosy. Nevertheless, the long-standing lack of a surveillance system prevents a real evaluation of endemicity. This is the first study to evaluate eradication of Leprosy on Reunion Island. The prevalence rate of less than one case per 10000 inhabitants is necessary, but not sufficient to claim eradication. Remaining active transmission of the disease is to be explored. The most widely used indicator of active transmission, the absence of new cases detected in children younger than 15 years of age, and the lack of focus of transmission, confirmed the eradication assumption. Improvements in quality of life, better housing conditions and lower promiscuity have played a key role in the reduction of autochthonous transmission. Active detection among relatives, systematic declaration and rapid treatment are the most effective way of preventing disabilities and further transmission of the disease. However, if elimination of leprosy is no longer a major public health problem on Reunion Island, the risk of reintroduction of the disease through immigration from endemic neighbouring countries is a real and continuing risk. Preventing resurgence is now the challenge.

## Introduction

Leprosy, also known as Hansen’s disease, is a chronic infectious disease caused by *Mycobacterium leprae*. Its most likely route of transmission is the upper respiratory tract, and it has a long incubation period [1]. The disease primarily affects the skin and peripheral nerves, causing sensory loss [2]. If not treated, it can cause progressive and permanent damage to the skin, nerves, limbs or eyes, and may lead to amputations and disabilities. Because of these visible symptoms, leprosy has always been strongly stigmatized, preventing patients to seek treatment. Leprosy can be cured using multidrug therapy (MDT), an association of different antibiotics including rifampin, dapsone and clofazimine [3].

Human leprosy has been documented for millennia and is probably the oldest human-specific infection [4]. The disease was distributed worldwide during the Middle Ages, but its prevalence has considerably decreased since MDT became available in the early 1980s [5] and national campaigns and disease surveillance systems were developed in most endemic countries. At the beginning of 2012, the registered prevalence of leprosy at global level was around 180,000 cases. The majority of new cases (95%) were reported from 16 countries. Some areas remain highly endemic, such as the Comoros and Mayotte in the Indian Ocean[6].

Reunion Island is a French overseas territory located in the South West Indian Ocean, 700 km to the East of Madagascar. Leprosy first arrived on Reunion Island in the early eighteenth century with African slaves and immigrants from Madagascar [7]. Leprosy continued to be a serious concern on Reunion Island until the 1960s, when about 148 patients were still followed in 1966 [8]; The disease was still endemic on Reunion Island until 1980 [9]. Improvements in the health care and living conditions of residents of the Island led to a significant decrease in the number of new cases of leprosy. However, since the prevalence of the illness on Reunion Island has been poorly documented due to the lack of an adequate surveillance system long preventing proper reporting of the illness, which means that it was impossible to know if the World Health Organisation’s goal to eradicate the disease (i.e., prevalence rate <1/10 000) has been truly achieved on Reunion Island.

In this context, the Regional Office of the French Institute for Public Health Surveillance (CIRE Indian Ocean) conducted in 2010 a retrospective study to collect information on all cases diagnosed between January 2005 and December 2010. This retrospective study showed that leprosy was still present on Reunion Island. A prospective surveillance system was then implemented in January 2011 [10].

The aim of the present study was to estimate the number of new cases of leprosy detected annually on Reunion Island between 2005 and 2013, describe the clinical features of patients and finally to evaluate eradication of leprosy on Reunion Island

## Methods

### Study design

This article is based on a descriptive study of new leprosy cases diagnosed between 2005 and 2013 on Reunion Island. The study was conducted retrospectively between 2005 and 2010 and prospectively between January 2011 and December 2013.

### Surveillance system and case identification

In 2010, as the lack of an adequate surveillance system made impossible to know if WHO objective for eradication was achieved, the CIRE Indian Ocean decided to conduct a retrospective study on all cases diagnosed in the last 5 years (2005–2010 period). This study involved health professionals who were in a position to collect diagnosed cases of leprosy. Private and hospital dermatologists, infectiologists and anti-tuberculosis centres were first informed about the study by letter and then contacted by telephone to report all cases of leprosy that occurred during this period by completing a standardized questionnaire. This retrospective study showed that leprosy was still present on Reunion Island and that the implementation of a prospective surveillance system was needed [10].

From January 2011, health professionals reported systematically all newly diagnosed cases using the same standardized questionnaire as in the retrospective study. In addition to clinicians, pathology laboratories are now requested to report the histologic diagnoses of new leprosy cases in order to ensure the completeness of data collection. All new leprosy patients must be sent for treatment to a referent physician in the anti-tuberculosis centre of the University Hospital of Reunion Island.

### Study population

The diagnosis of leprosy is based on the World Health Organization’s criteria: “patient presenting skin lesion consistent with leprosy and with definite sensory loss, with or without thickened nerves and/or positive skin smears” [11]. In our study, all patients had a skin biopsy and/or a nose and ear smear to confirm the diagnosis, except for 2 patients who had been diagnosed several years earlier and presented clinical features of relapse. When the bacteriological index was positive on the biopsy or nose and ear smear, the patient was classified as multibacillary. Patients showing clinical manifestations of leprosy but negative smears were classified as paucibacillary.

### Collected data and statistical analysis

The retrospective study was based on data collected at CIRE Indian Ocean. The prospective study was based on systematic reporting.

All the information was collected using a standardized questionnaire. The following data were collected for each patient: socio-demographic data (age, country of birth, country of residence, sex, profession), type of leprosy according to WHO classification (multibacillary for patients with positive smears and paucibacillary for patients with negative smears), and clinical data (method of diagnosis, degree of disability evaluated at the time of reporting, i.e., before treatment). Disability was classified according to the WHO grading system (grade 1: decrease or loss of sensibility in the eyes, hands and/or feet; grade 2: Disability or deformity in the eyes, hands and/or feet) [11]. All patient data were anonymized.

Quantitative variables were expressed as mean and standard deviation or median and interquartile range. Qualitative variables were expressed as proportions and 95% confidence interval. We performed separate analyses for each period of collection. Registered prevalence was calculated by dividing the number of yearly cases reported by Reunion Island’s population that year, and multiplying that number by 10,000.

## Results

Results are summarized in Table 1. From January 2005 to December 2013 (9 years), 25 new cases of leprosy were diagnosed on Reunion Island. During the first period of our study (2005–2010) 18 cases were diagnosed and 7 during the second period (2011–2013). The median age of patients at the time of diagnosis was 48.2 years in the first period versus 44.3 years in the second; moreover, male patients were predominant in the entire period (68%, 17/25). Only 1 child under 15 years of age was diagnosed with leprosy in the first period, and none in the second. This child was born in the Comoros and had recently migrated to Reunion Island; hence he was probably contaminated in the Comoros.

**Table 1:** Main characteristics of the leprosy cases identified on Reunion Island between 2005 and 2013.

Periods	[2005–2010]	[2011–2013]	[2005–2013]
Variables (units)	(n = 18)	(n = 7)	(n = 25)
**Socio-demographic features**			
Age at diagnosis (years)			
mean ± sd	48.2 ± 22.5	44.3 ± 21.3	47.1 ± 21.7
Min—Max	(8–77)	(22–76)	(8–77)
Patients under 15 years-old			
Nb (%)	1 (5.6%)	-	1 (4.0%)
Sex ratio M/F			
%	77.8/ 22.2	42.9/ 57.1	68.0/ 32.0
**Geographic features** Nb (%)			
Country of birth			
Reunion Island	10 (55.6%)	2 (28.6%)	12 (48.0%)
Comoros	4 (22.2%)	2 (28.6%)	6 (24.0%)
Mayotte	3 (16.7%)	3 (42.9%)	6 (24.0%)
Madagascar	1 (5.6%)	-	1 (4.0%)
autochthonous/ imported			
Confirmed autochthonous cases	10 (55.6%)	2 (28.6%)	12 (48.0%)
Possible autochthonous cases	3 (16.7%)	-	3 (12.0%)
Imported cases	5 (27.8%)	5 (71.4%)	10 (40.0%)
**Clinical features** Nb (%)			
**Diagnosis method** (and/or)			
Skin biopsy	15 (83.3%)	6(85.7%)	21 (84.0%)
Smears (ear or nose)	4 (22.2%)	-	4 (16.0%)
Clinical features only^1^	1 (5.6%)	1 (14.3%)	2 (8.0%)
**Microbiological classification**^2^			
paucibacillary	2 (11.1%)	5 (71.4%)	7 (28.0%)
multibacillary	16 (88.9%)	2 (28.6%)	18 (72.0%)
**Clinical classification**			
No skin lesion	-	1 (14.3%)	1 (4.0%)
≤ 5 skin lesions	2 (11.1%)	4 (57.1%)	6 (24.0%)
> 5 skin lesions	16 (88.9%)	2 (28.6%)	18 (72.0%)
**Clinical forms**^3^			
Undetermined	-	1 (14.3%)	1 (4.0%)
Tuberculoid	2 (11.1%)	4 (57.1%)	6 (24.0%)
Lepromatous	16 (88.9%)	2 (28.6%)	18 (72.0%)
Borderline	-	-	-
**Physical disability at the time of reporting**			
None	6 (33.3%)	3 (42.9%)	9 (36.0%)
Grade 1 localized on hands and/or feet	6 (33.3%)	2 (28.6%)	8 (32.0%)
Grade 2 localized on hands and/or feet	4 (22.2%)	2 (28.6%)	6 (24.0%)
unspecified	2 (11.1%)	-	2 (8.0%)

yo = years-old

F/M = female/male

sd = standard deviation

^1^ These 2 patients have been diagnosed several years earlier. Clinical features of relapse were considered sufficient.

^2^ based on absence (paucibacillary) or presence (multibacillary) of mycobasterium leprae on skin smears

^3^ According to Ridley-Jopling classification

Among the 25 new cases, 12 are Reunion Island residents who never lived outside the Island, and are therefore considered to be autochthonous patients (“Confirmed autochthonous cases”). There were 10 new autochthonous cases in the first period (6 years: 2005–2010), and only 2 in the second (3 years: 2011–2013). 6 of these autochthonous patients lived in the same area of Saint-Louis, a popular city in the southwest of the island that constituted a focus of transmission.

Among the 13 patients born or having resided outside Reunion Island (Comoros, Mayotte or Madagascar), 10 cases arrived on Reunion Island less than 5 years before the diagnosis. Considering the mean duration of leprosy incubation (about 5 years according to the WHO), these cases were then considered as imported cases. 2 other cases arrived on Reunion Island 9 and 12 years before the diagnosis; and for the last one the date of arrival on Reunion Island was not specified. Those 3 cases were doubtful and were then, taking the most pessimistic option, considered as possible autochthonous cases.

Skin biopsy was largely available on the island; the majority of diagnoses were therefore made with skin biopsy (84%, 21/25). However, some patients had a complementary ear and nose biopsy to classify the case based on WHO criteria. The multibacillary form was predominant (72%, 18/25). The rate of new cases with grade 2 disability was 24% (6/25), and 56% (14/25) of patients had a grade 1 or 2 disability at the time of detection.

## Discussion

Our study show that the number of cases of leprosy reported between 2005 and 2013 is compatible with the eradication of the disease on Reunion Island.

### Indicators of active transmission

Although leprosy is now diagnosed in less than 1/10 000 inhabitants on Reunion Island, it is important to follow indicators of active transmission in the community. Our study shows that few autochthonous cases of patients are still present. These autochthonous cases are proportionally fewer in the second period than in the first with no more focus of transmission which suggests that autochthonous transmission on the Island is disappearing. Interestingly, no cases of patient under 15 years of age were detected in the second period of the study, indicating that there has been no active transmission for the last 3 years. These 2 keys indicators supports that autochthonous transmission of leprosy has stopped on Reunion Island.

However, regarding clinical features of patients, a high rate of disability at the time of diagnosis has been reported for 24% of the patients (grade 2 disability), which is indicative of late detection. This might be explained by general practitioners’ poor knowledge of the disease. Following the study, communication has been performed to renew GPs awareness of the risk of leprosy on Reunion Island.

### Limitations

Our study presents some limitations. Indeed, the possibility that some misdiagnosed or undeclared cases cannot be excluded. Furthermore, the prospective period (3 years) is too short to conclude that the illness has been lastingly eradicated from the island. This result has to be re-evaluated regularly.

**Fig 1:**
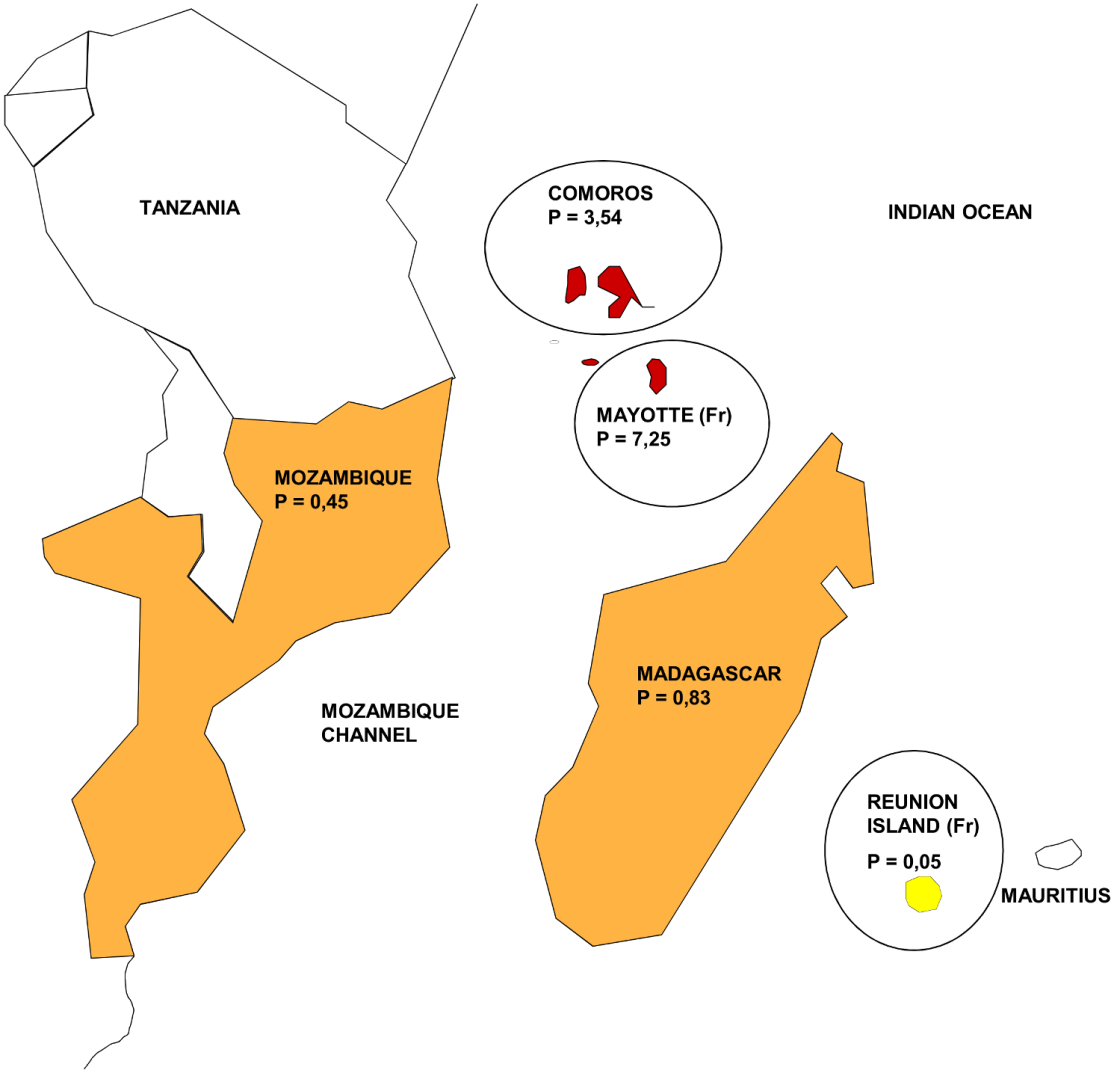
Prevalence rate of Leprosy in Indian Ocean in 2014. P (Prevalence rate) = number of cases/ 10 000 inhabitants; Fr = French Oversea Departments; For Tanzania and Mauritius: No data available; For Mozambic, Madagascar and Comoros: data from the Weekly Epidemiological record, Sept 2015; For Reunion and Mayotte islands: data from the ALLF (association of francophone leprologists) bulletin N°30, June 2015; Red = highly endemic; orange = endemic; yellow = eradicated.

In our opinion, this favourable evolution can be explained by one major historical reason. Reunion Island was the first overseas territory to become an administrative French department in 1946; as a result, the island has benefitted from the French public health system for more than 50 years. For example, dermatologic consultation has been available on the entire territory for over 3 decades. The skin biopsy became the gold standard for diagnosis in routine. This important access to dermatologists has played a large role in the early detection of new cases, which is the key for preventing aggravation and transmission [12,13]. In addition, improvements in quality of life, better housing conditions and lower promiscuity have played an important role in the reduction of autochthonous transmission. Indeed household and dwelling contact are among the most important risk factors for transmission [14]. By contrast, Mayotte Island has just gained the same administrative status as Reunion Island, and the living conditions of the majority of its inhabitants are still rudimentary, with small homes for large families and poor access to medical care. This situation may well explain partly why leprosy is still endemic in Mayotte [15]. Moreover, given their long-standing historical ties, Mayotte has seen an important number of imported cases from the Comoros, where the disease is also endemic (Fig 1). In fact, Mayotte and the Comoros have the highest prevalence rate of leprosy of the South Indian Ocean area.

Lastly, the implementation of a tuberculosis and leprosy control program—which includes active surveillance, early declaration of new cases prompting the screening of household contacts and rapid access to MDT,—can also explain the progressive eradication of the disease.

### Risk of resurgence

However, Reunion Island remains highly exposed to resurgence. Indeed, the reintroduction of the disease through immigration from endemic neighbouring countries such as the Comoros, Mayotte or Madagascar is a real and continuing risk. This risk is illustrated by the prevalence rates of Leprosy in Indian Ocean in 2014 (Fig 1). Main immigration in Reunion Island comes from Madagascar, Comoros, and Mayotte. In 2014, Comoros and Mayotte were still highly endemic with prevalence rates of 3.54 and 7.25/10 000 inhabitants. With a rate of 0.83/10000 inhabitants, Madagascar is also among the endemic countries. Constant vigilance should then be maintained to assure that the disease does not reappear in the community.

### Conclusion

Leprosy is no longer a major public health problem on Reunion Island, as indicated by the low prevalence rate and the absence of active transmission. Improvements in living conditions and access to health care meeting French metropolitan standards have put an end to autochthonous transmission. However, given the significant influx of migrants from leprosy-endemic neighbouring countries, the risk of resurgence of the disease and of renewed autochthonous transmission is real. In conclusion, our experience shows that “active detection, systematic declaration and rapid treatment” are the 3 key measures to obtain eradication of leprosy in a community. In our opinion, those measures must be maintained to consolidate eradication.

## Supporting Information

S1 Checklist STROBE for observational studies. (DOCX)
