# Supplementary material for: Leprosy on Reunion Island, 2005-2013: Situation and Perspectives
Source: PLoS Negl Trop Dis. 2016 Apr 15;10(4):e0004612. doi: 10.1371/journal.pntd.0004612 (PMC4833340; doi:10.1371/journal.pntd.0004612)
Supplement: S1 Checklist — (DOCX) [file pntd.0004612.s001.docx]

STROBE Statement—checklist of items that should be included in reports of observational studies

|  | Item No. | Recommendation | Page  No. | Relevant text from manuscript |
| --- | --- | --- | --- | --- |
| **Title and abstract** | 1 | (*a*) Indicate the study’s design with a commonly used term in the title or the abstract | 2 | Abstract – background : line 26 |
|  |  | (*b*) Provide in the abstract an informative and balanced summary of what was done and what was found | 2 | Abstract – Methodology /Principal Findings: lines  29-37 |
| Introduction | | | |  |
| Background/rationale | 2 | Explain the scientific background and rationale for the investigation being reported | 4-5 | Introduction : lines  62-91 |
| Objectives | 3 | State specific objectives, including any prespecified hypotheses | 5 | Introduction : lines  92-94 |
| Methods | | | |  |
| Study design | 4 | Present key elements of study design early in the paper | 5 | Methods – study design : lines 99-101 |
| Setting | 5 | Describe the setting, locations, and relevant dates, including periods of recruitment, exposure, follow-up, and data collection | 5-6 | Methods – Surveillance system and case identification: lines 103-115 |
| Participants | 6 | Give the eligibility criteria, and the sources and methods of selection of participants. Describe methods of follow-up | 6 | Methods – study population: lines 118-124 |
| Variables | 7 | Clearly define all outcomes, exposures, predictors, potential confounders, and effect modifiers. Give diagnostic criteria, if applicable | 6 | Methods – study population: lines 118-124 |
| Data sources/ measurement | 8* | For each variable of interest, give sources of data and details of methods of assessment (measurement). Describe comparability of assessment methods if there is more than one group | 6 | Methods – Collected data and Statistical analysis : lines 126-128 |
| Bias | 9 | Describe any efforts to address potential sources of bias | 7 | Methods – Collected data and Statistical analysis : lines 128-135 |
| Study size | 10 | Explain how the study size was arrived at |  | *Not applicable* |

Continued on next page

| Quantitative variables | 11 | Explain how quantitative variables were handled in the analyses. If applicable, describe which groupings were chosen and why | 7 | Methods – Collected data and Statistical analysis : line 138 |
| --- | --- | --- | --- | --- |
| Statistical methods | 12 | (*a*) Describe all statistical methods, including those used to control for confounding | 7 | Methods – Collected data and Statistical analysis : lines 136-140 |
|  |  | (*b*) Describe any methods used to examine subgroups and interactions |  | *Not applicable* |
|  |  | (*c*) Explain how missing data were addressed | 8 | Results : line 162 |
|  |  | (*d*) *Cohort study*—If applicable, explain how loss to follow-up was addressed  *Case-control study*—If applicable, explain how matching of cases and controls was addressed  *Cross-sectional study*—If applicable, describe analytical methods taking account of sampling strategy |  | *Not applicable* |
|  |  | (*e*) Describe any sensitivity analyses |  | *Not applicable* |
| Results | | | | |
| Participants | 13* | (a) Report numbers of individuals at each stage of study—eg numbers potentially eligible, examined for eligibility, confirmed eligible, included in the study, completing follow-up, and analysed | 7 | Results : lines 144-146 |
|  |  | (b) Give reasons for non-participation at each stage |  | *Not applicable* |
|  |  | (c) Consider use of a flow diagram |  | *Not applicable* |
| Descriptive data | 14* | (a) Give characteristics of study participants (eg demographic, clinical, social) and information on exposures and potential confounders | 7 | Results : lines 146-149 |
|  |  | (b) Indicate number of participants with missing data for each variable of interest | 8 | Results : lines 161-162 |
|  |  | (c) *Cohort study*—Summarise follow-up time (eg, average and total amount) |  | *Not applicable* |
| Outcome data | 15* | *Cross-sectional study—*Report numbers of outcome events or summary measures | 8 | Results : lines 152-156 |
| Main results | 16 | (*a*) Give unadjusted estimates and, if applicable, confounder-adjusted estimates and their precision (eg, 95% confidence interval). Make clear which confounders were adjusted for and why they were included | 8 | Results : lines 152-169 |
|  |  | (*b*) Report category boundaries when continuous variables were categorized | 9 | Table 1 |
|  |  | (*c*) If relevant, consider translating estimates of relative risk into absolute risk for a meaningful time period |  |  |

Continued on next page

| Other analyses | 17 | Report other analyses done—eg analyses of subgroups and interactions, and sensitivity analyses | 9 | Table 1 |
| --- | --- | --- | --- | --- |
| Discussion | | | | |
| Key results | 18 | Summarise key results with reference to study objectives | 10 | Discussion – lines 182-183 |
| Limitations | 19 | Discuss limitations of the study, taking into account sources of potential bias or imprecision. Discuss both direction and magnitude of any potential bias | 10-11 | Discussion – limitation : lines 198-201 |
| Interpretation | 20 | Give a cautious overall interpretation of results considering objectives, limitations, multiplicity of analyses, results from similar studies, and other relevant evidence | 10-11 | Discussion – limitation : lines 198-201 |
| Generalisability | 21 | Discuss the generalisability (external validity) of the study results |  | Discussion – lines 202-240 |
| Other information | |  | | |
| Funding | 22 | Give the source of funding and the role of the funders for the present study and, if applicable, for the original study on which the present article is based |  | *Not applicable* |

*Give information separately for cases and controls in case-control studies and, if applicable, for exposed and unexposed groups in cohort and cross-sectional studies.

**Note:** An Explanation and Elaboration article discusses each checklist item and gives methodological background and published examples of transparent reporting. The STROBE checklist is best used in conjunction with this article (freely available on the Web sites of PLoS Medicine at http://www.plosmedicine.org/, Annals of Internal Medicine at http://www.annals.org/, and Epidemiology at http://www.epidem.com/). Information on the STROBE Initiative is available at www.strobe-statement.org.
